# Atraumatic bleeding of the subclavian artery 20 years after surgical treatment of pneumothorax

**DOI:** 10.1186/s13019-020-1052-2

**Published:** 2020-01-08

**Authors:** Kentaro Miura, Nobutaka Kobayashi

**Affiliations:** Department of Thoracic Surgery, Japanese Red Cross Society Nagano Hospital, 5-22-1 Wakasato, Nagano, 380-8582 Japan

**Keywords:** Atraumatic, Subclavian artery, Bleeding, Pneumothorax, Stent graft

## Abstract

**Background:**

Bleeding of the subclavian artery is a fatal condition. Adhesion between the pleura and staple line may develop after surgical treatment of pneumothorax, and collateral arteries often develop from the subclavian artery toward the adhesion at the lung apex; however, atraumatic tearing and bleeding of these collateral arteries into the extrapleural and intrathoracic cavities is rare.

**Case presentation:**

A 70-year-old man visited the hospital for evaluation of left chest pain. Contrast-enhanced chest computed tomography showed a huge tumor in the left apex of the lung. It was suspected to be an extrapleural huge hematoma, and it ruptured into the thoracic cavity. Bleeding from the left subclavian artery was suspected; therefore, emergency angiography was performed. Angiography showed some collateral circulation from the left subclavian artery to the apex of the left lung. Distal and proximal bleeding points were identified. The distal bleeding point was embolized using coils. The proximal bleeding point was blown out, and stents were placed in the left subclavian artery. He had undergone pneumothorax surgery 20 years previously, and the present bleeding episode was strongly suspected to be associated with that surgery. The collateral circulation from the subclavian artery could have developed because of post-pneumothorax inflammation, eventually rupturing and bleeding into the extrapleural space.

**Conclusions:**

This report described an important case of atraumatic subclavian artery bleeding considered to have been caused by surgical treatment of pneumothorax 20 years previously. Emergency angiography and percutaneous stent placement or coil embolization should be considered first in such cases.

## Background

Bleeding of the subclavian artery is a fatal condition. Many reports of subclavian artery injury describe a direct association with first rib fractures [1] [2] [3], and some describe complication with von Recklinghausen disease [4]. Bleeding may be controlled by either open repair (median sternotomy, lateral thoracotomy) or endovascular repair.

Adhesion between the pleura and staple line may develop after surgical treatment of pneumothorax. In such cases, collateral arteries often develop from the subclavian artery toward the adhesion at the lung apex; however, atraumatic tearing and bleeding of these collateral arteries into the extrapleural and intrathoracic cavities is rare.

We herein report a case of atraumatic subclavian artery bleeding that was suspected to be associated with surgical treatment of pneumothorax performed 20 years previously.

## Case presentation

**Fig. 1:**
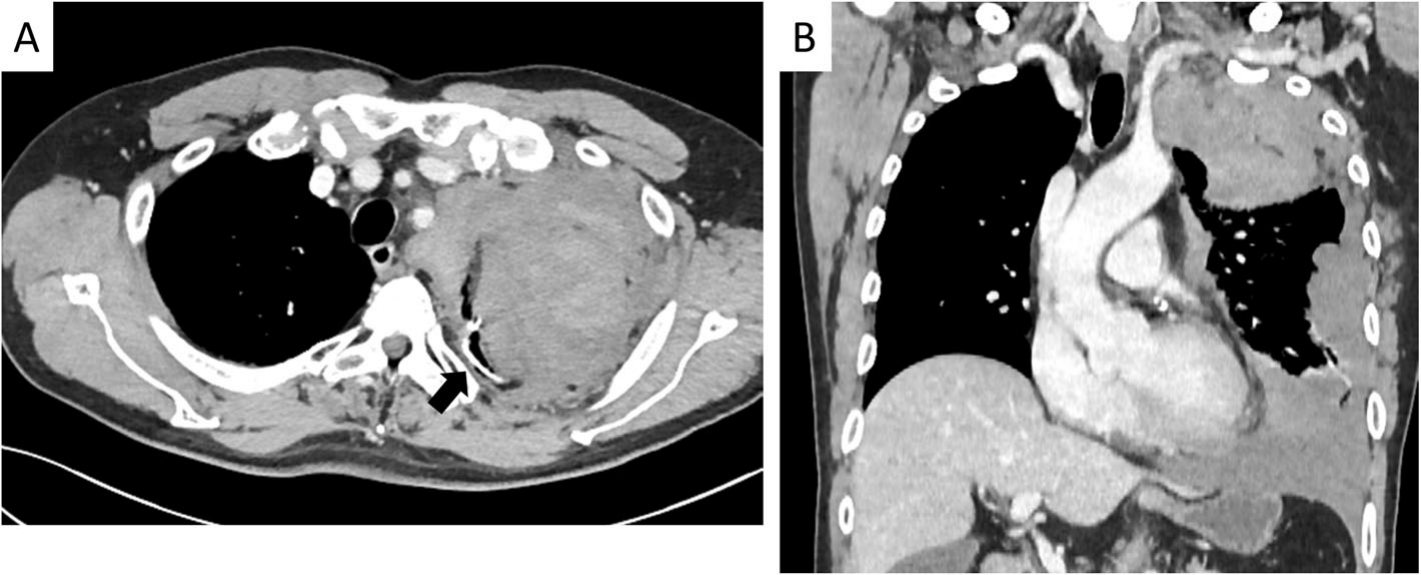
**a**, **b** Chest computed tomography showed an extrapleural hematoma and hemothorax. The staple line of the pneumothorax surgery 20 years previously was confirmed (arrow)

A 70-year-old man visited another hospital because he had experienced left chest pain for half a day. He had a history of atrial fibrillation and had taken an anticoagulant for many years. He had undergone VATS bullectomy for pneumothorax 20 years previously and the period of post-operative chest tube drainage had been long because of refractory air leakage. Plueodesis had not been performed. He had no history of hereditary diseases such as von Recklinghausen disease. Chest computed tomography showed a giant tumor in the apex of the lung and staple line of the pneumothorax surgery, however, he came home because his vital sign was stable. He returned to the hospital the next day because his left chest pain had worsened. Contrast-enhanced chest computed tomography showed left hemothorax in addition to the superior sulcus tumor and staple line of the pneumothorax surgery (Fig. 1a, b). The huge tumor in the apex of the lung was suspected to be an extrapleural huge hematoma, and it ruptured into the pleural cavity. He was raced to our hospital and a chest tube was inserted into the left pleural cavity, resulting in drainage of a large volume of bloody fluid. The patient then developed shock, and a massive blood transfusion was started.

**Fig. 2:**
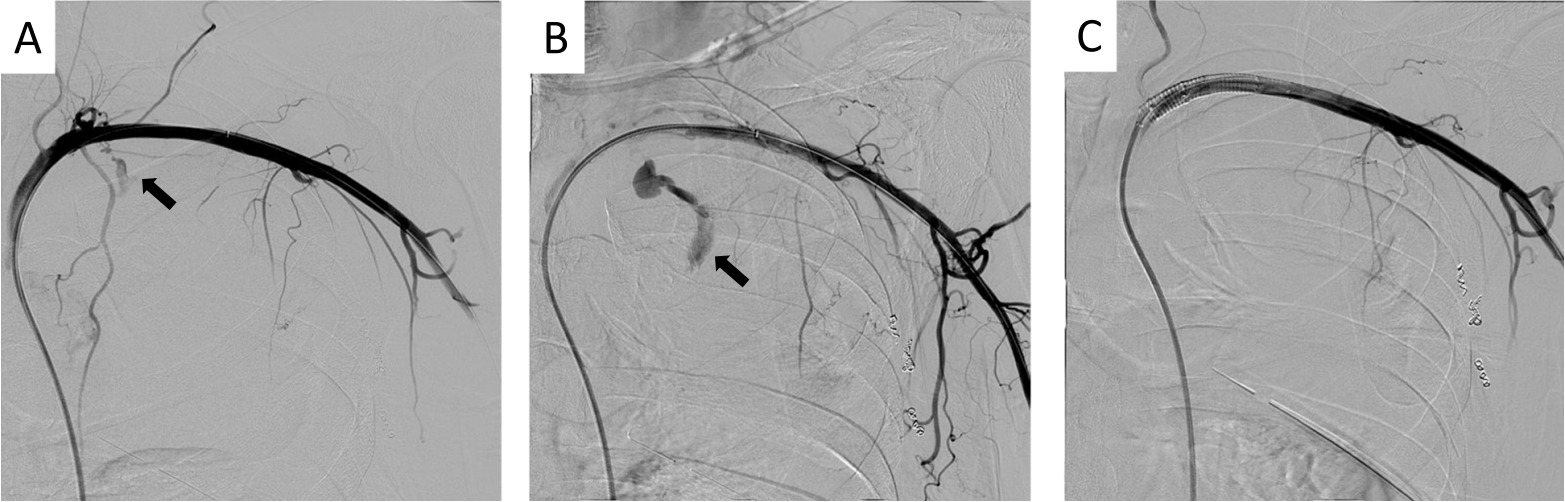
Angiography of the left subclavian artery. Some collateral arteries had developed toward the apex of the left lung. **a**, **b** The arrow indicates ejective bleeding from the proximal to left subclavian artery. **c** The bleeding was stopped using stents

Bleeding from the left subclavian artery was suspected, and emergency angiography via the right femoral artery was performed. Angiography showed some collateral arteries from the left subclavian artery to the apex of the left lung. Distal and proximal bleeding points were identified. The distal bleeding point was embolized using coils. The proximal bleeding point (Fig. 2a, b) was blown out, and 8.0- × 50-mm stent grafts (GORE VIABAHN; W. L. Gore & Associates, Newark, DE, USA) were placed in the left subclavian artery with careful attention to occlusion of the vertebral artery (Fig. 2c). The shock and bleeding continued during the angiography, and the massive blood transfusion was continued. After placement of the stents, the patient’s vital signs stabilized. The amount of bleeding was 2000 ml. The chest tube was removed 4 days after treatment, and he was discharged without sequelae.

## Discussion

This case illustrates two important findings. First, atraumatic bleeding from the subclavian artery to the extrapleural or thoracic cavity is rare; however, it is a critical situation that requires rapid bleeding control. We successfully stopped the bleeding by emergency angiography via the right femoral artery and percutaneous stent placement. Although we initially considered surgical hemostasis, we expected that it would be difficult to find the bleeding point while removing the hematoma, and the risk of massive bleeding was high. Second, our patient’s bleeding was strongly suspected to be associated with the pneumothorax surgery performed 20 years previously. The collateral arteries from the subclavian artery could have developed secondary to pneumothorax-induced inflammation; after these vessels ruptured and bled into the extrapleural cavity, the pleura became compromised and hemothorax developed.

A few reports have described bleeding from the subclavian artery. Santin et al. [4] reported subclavian artery rupture in a patient with von Recklinghausen disease. They successfully stopped the bleeding using an 8-mm × 5-cm endoprosthesis stent graft via the brachial sheath, as in our case.

Some reports have also described open thoracotomy. Tennyson et al. [2] reported traumatic subclavian artery rupture that was successfully repaired by thoracotomy and direct suturing. However, we considered that it would be difficult to perform open thoracotomy in our patient because the bleeding point was unclear. Additionally, his shock and life-threatening condition necessitated a more rapid approach. Thus, emergency angiography with percutaneous stent placement rather than thoracotomy under general anesthesia was chosen in this case.

Angiography showed collateral arteries from the left subclavian artery to the left apical portion of the lung. In this case, the period of post-operative chest tube drainage had been long because of refractory air leakage after bullectomy. The collateral circulation from the subclavian artery could have developed because of post-pneumothorax inflammation, eventually rupturing and bleeding into the extrapleural space. We considered that these adhesions and collateral arteries had been ruptured by coughing or similar stimulation, and administration of anticoagulant therapy promoted the bleeding. Terada et al. [5] reported a case of intractable hemoptysis by the branches of the right subclavian artery caused by an aspergilloma, and the patient was successfully treated with arterial coil embolization. The authors considered that these abnormal branches were caused by pleural adhesion to the aspergilloma. Thus, inflammation induced by surgical treatment of the pneumothorax could have caused the adhesion and development of collateral arteries from the subclavian artery in our case. It is unclear how often like this fatal bleeding will happen in patient who had performed pneumothorax surgery or pleurodesis, however, the possibility of like this bleeding should be kept in mind.

## Conclusion

In conclusion, this report described an important case of atraumatic subclavian artery bleeding considered to have been caused by surgical treatment of pneumothorax 20 years previously. Emergency angiography and percutaneous stent placement or coil embolization should be considered first in such cases.

## Data Availability

The data used in this report are available from the corresponding author on reasonable request.
